# Trisomy 8, a Cytogenetic Abnormality in Myelodysplastic Syndromes, Is Constitutional or Not?

**DOI:** 10.1371/journal.pone.0129375

**Published:** 2015-06-12

**Authors:** Sílvia Saumell, Francesc Solé, Leonor Arenillas, Julia Montoro, David Valcárcel, Carme Pedro, Carmen Sanzo, Elisa Luño, Teresa Giménez, Montserrat Arnan, Helena Pomares, Raquel De Paz, Beatriz Arrizabalaga, Andrés Jerez, Ana B. Martínez, Judith Sánchez-Castro, Juan D. Rodríguez-Gambarte, José M. Raya, Eduardo Ríos, María Rodríguez-Rivera, Blanca Espinet, Lourdes Florensa

**Affiliations:** 1 Laboratori de Citologia Hematològica i Citogenètica Molecular, Servei de Patologia, Hospital del Mar, Barcelona, Spain; 2 GRETNHE, Cancer Research Program, IMIM (Hospital del Mar Medical Research Institute), Barcelona, Spain; 3 Department of Medicine, Medicine Faculty, Universitat Autònoma de Barcelona, Bellaterra, Spain; 4 Institut de Recerca Contra la Leucèmia Josep Carreras, Cytogenetics Platform, Badalona, Spain; 5 Servicio de Hematología, Hospital Vall d’Hebrón, Barcelona, Spain; 6 Servei de Hematologia Clínica, Hospital del Mar, Barcelona, Spain; 7 Servicio de Hematología, Hospital Central de Asturias, Oviedo, Spain; 8 Servei d’Hematologia, Hospital Universitari Joan XXIII, Tarragona, Spain; 9 Servei d’Hematologia, Hospital Duran i Reynals, Institut Català d’Oncologia, L’Hospitalet del Llobregat, Spain; 10 Servicio de Hematología, Hospital Universitario de La Paz, Madrid, Spain; 11 Servicio de Hematologia, Hospital Universitario de Cruces, Baracaldo, Spain; 12 Servicio de Hematología, Hospital Morales Meseguer, Murcia, Spain; 13 Servei d’ Hematologia, Hospital Universitari Arnau de Vilanova, Lleida, Spain; 14 Servicio de Hematología, Hospital Universitario Ramon y Cajal, Madrid, Spain; 15 Servicio de Hematología, Hospital Universitario de Canarias, La Laguna,Tenerife, Spain; 16 Sevicio de Hematologia, Hospital Universitario de Valme, Sevilla, Spain; Queen's University Belfast, UNITED KINGDOM

## Abstract

Isolated trisomy 8 is not considered presumptive evidence of myelodysplastic syndrome (MDS) in cases without minimal morphological criteria. One reason given is that trisomy 8 (+8) can be found as a constitutional mosaicism (cT8M). We tried to clarify the incidence of cT8M in myeloid neoplasms, specifically in MDS, and the diagnostic value of isolated +8 in MDS. Twenty-two MDS and 10 other myeloid neoplasms carrying +8 were studied. Trisomy 8 was determined in peripheral blood by conventional cytogenetics (CC) and on granulocytes, CD3+ lymphocytes and oral mucosa cells by fluorescence *in situ* hybridization (FISH). In peripheral blood CC, +8 was seen in 4/32 patients. By FISH, only one patient with chronic myelomonocytic leukemia showed +8 in all cell samples and was interpreted as a cT8M. In our series +8 was acquired in all MDS. Probably, once discarded cT8M by FISH from CD3+ lymphocytes and non-hematological cells, +8 should be considered with enough evidence to MDS.

## Introduction

Myelodysplastic syndromes (MDS) are a heterogeneous group of acquired clonal hematopoietic stem cell disorders with increased risk of acute myeloid leukemia (AML) development. Diagnosis of MDS remains among the most challenging of the myeloid neoplasms and is based on the presence of cytopenia(s), dysplasia in one or more myeloid lineages and less than 20% bone marrow (BM) or peripheral blood (PB) blasts [1,2]. Around 50% of MDS cases presented clonal cytogenetic abnormalities [2]. Trisomy 8 (+8) is the most common chromosome gain in MDS and is present in 5–7% of them [3]. MDS patients with isolated +8 are included in the MDS intermediate cytogenetic risk group according to the new revised IPSS (IPSS-R) [4]. Nevertheless, in contrast to other recurring chromosomal alterations, the presence of +8 as the sole cytogenetic abnormality is not considered definitive evidence for MDS in the absence of morphological criteria [2]. Since trisomy 8 was found as a constitutional mosaicism (cT8M) in healthy people, it was not considered a tumour marker by some authors [5]. However, the incidence of cT8M referred is very low; Nielsen and Wohlert detected one case of cT8M among approximately 35000 live births [6], and Seghezzi et al. found two cases out of 40140 [7]. In addition, some studies suggested that +8 could be present as a cT8M in myeloid malignancies [7–10]^,^ and Maserati et al. reported that +8 is constitutional in 15–20% of MDS and acute leukemia [9]. We have analyzed the presence of +8 in granulocytes and CD3+ lymphocytes from PB, as well as in oral mucosa cells from patients diagnosed with MDS carrying +8, in order to clarify the incidence of cT8M in MDS and try to provide a precise diagnostic and prognostic value for isolated +8, especially in cases where there is a degree of doubt.

## Methods

A total of 32 patients with +8 were studied from different Spanish hospitals belonging to the *Grupo español de síndromes mielodisplásicos* (GESMD): 22 diagnosed with MDS and 10 of other myeloid neoplasms. The latter group included four patients with myelodysplastic/ myeloproliferative neoplasm [two chronic myelomonocitic leukemia (CMML) and two refractory anemia with ring sideroblasts and thrombocytosis (RARS-T)] and six patients with AML. Five of the MDS and two of the AML patients had additional cytogenetic alterations to +8 on the bone marrow karyotype. One of the AML had a tetrasomy 8. Furthermore, we also studied 20 healthy controls (12 women and 8 men), with ages ranged between 20–60 years.

### Blood Samples

Lymphocytes and granulocytes were isolated from 30mL of PB using standard cell separation protocols. CD3+ cells were isolated from mononuclear cells by immunomagnetic beads (MiltenyiBiotec, Germany). Afterwards, CD3+ cells, as well as granulocytes, were fixed with Carnoy fixative solution (3:1 methanol to acetic acid), and spread on independent slides for fluorescence *in situ* hybridization (FISH) studies. The decision to study CD3+ cells was based on the discarted involvement of them in MDS [11–16], their practical accessibility, and the recommendations of other authors for germline analisis in SNP and sequencing studies [17–19].

### Oral Mucosa

The oral mucosa was scraped with a sterile cotton swab. Four smears were made by scattering mucosa cells of the swabs over slides. The samples were fixed 10 min in Carnoy solution. Once dried, slides were treated with acetic acid solution (3:2 acetic acid to methanol) at 45°C for 40 min, following with a 10 min digestion in 0.005% pepsin solution (Sigma Aldrich, St Louis, MO) at 37°C, and ending with a dehydratation in 70%, 80% and 100% ethanol wash series.

### Karyotype Analysis

Metaphase staining chromosome analysis using phytohemagglutinin (PHA) stimulated cultures of PB were carried out by G-banding technique. At least 15 metaphases were analyzed for each patient. The analysis and nomenclature of the chromosomes were based on International System for Human Cytogenetic Nomenclature (ISCN) of 2013 [20].

### Fluorescence *in situ* Hybridization (FISH)

The centromeric 8 spectrum-orange DNA probe (CEP 8, Vysis, Downers Grove, IL) was applied to CD3+ lymphocytes, granulocytes and oral mucosa cells slides. The hybridization was performed overnight at 37°C. After washing, slides were counterstained with diaminophenylindole (DAPI II). The results of the hybridization were evaluated in a fluorescence microscope. If three signals of the same size and intensity were separated by at least one domain, +8 was considered. Following the European Cytogeneticists Association Specific Constitutional Guidelines [21], +8 mosaicism was assessed in 200 nuclei for CD3+ lymphocytes and granulocytes, and a minimum of 30 mucosa cells were analyzed. According to our laboratory, cutoff points for PB samples as well as for oral mucosa cells were 5%.

The study was carried out in accordance with the biomedical Helsinki Declaration of research guidelines and was approved by the *Comité Ético de Investigación Clínica (CEIC) Parc de Salut Mar*. All participants provided their written informed consent to participate in the study.

### Statistical analysis

Overall survival (OS) and time to AML transformation of patients with MDS and +8 were calculated. They were defined to be the time from the MDS diagnosis to death or last follow-up and to development of AML, respectively. Kaplan-Meier method was used to evaluate OS and AML transformation. Data analysis was performed using the R software package (version 3.1.1; R Foundation for Statistical Computing, Vienna, Austria).

## Results

The patient characteristics are shown in Table 1. Among 22 patients diagnosed with MDS and +8, 17 cases had isolated +8 on BM karyotype at diagnosis, and five had also other additional alterations. Cytogenetic analysis of PB PHA-stimulated cultures revealed +8 in 3 out of 22 patients in 5% to 65% of cells. Using FISH, trisomy 8 was observed in 3% to 74% of granulocytes from all 18 patients studied (4 patients were not studied for extremely neutropenia). Two of them were not considered positive for not reaching our cut off. For CD3+ cells samples, trisomy 8 was seen in 5 out of 22 patients. However, only 4 of them showed trisomy 8 over 5% (6% to 20%). Probably, those cells with +8 detected in CD3+ isolated samples were monocytes due to contamination during cellular isolation (CD3+ cell purity being 76 to 91.1%). None of the oral mucosa cell slides from 20 patients that could be analyzed showed +8, the other two cases could not be analyzed for unsuccessful hybridization.

**Table 1:** Patient Characteristics.

				FLUORESCENCE IN SITU HYBRIDIZATION				
				CD3+ LYMPHOCYTES		GRANULOCYTES	MUCOSA	
	WHO	BONE MARROW KARYOTYPE	PB KARYOTYPE (PHA)	% cells with +8	% of purity	% cells with +8	% cells with +8	N° of cells analyzed
** **	**MDS**	** **	** **	** **	** **	** **	** **	** **
1	RA	46,XX,del(5)(q13q33)[10]/47,sl,+8[3]/48,sld1,+22[4]/47,XX,+8[5]/46,XX[4]	46,XX[20]	0	96	20	0	46
2	RA	47,XY,+8[4]/46,XY[12]	46,XY[15]	0	96	3	0	100
3	RCUD	47,XX,+8[11]/46,XX[9]	46,XX[20]	0	88	74	0	75
4	RCMD	47,XX,+8[10]/46,XX[10]	47,XX,+8[5]/46,XX[15]	6	91.1	-	0	100
5	RCMD	47,XY,del(5)(q15q33),+8[20]	46,XY[15]	0	95	-	0	100
6	RCMD	47,XY,+8[10]/46,XY[10]	46,XY[15]	7	86	69	0	100
7	RCMD	47,XY,+8[7]/46,XY[13]	46,XY[15]	0	85	-	0	100
8	RCMD	47,XY,+8[15]/46,XY[5]	48,XY,+8,+21[1]/46,XY[19]	20	86	63	0	100
9	RCMD	47,XY,+8[16]/46,XY[4]	46,XY[20]	0	90	30	0	73
10	RCMD	47,XX,+8[20]/48,sl,+8[1]/46,XX[7]	46,XX[15]	0	92	31	-	-
11	RCMD	47,XX,+8[5]/46,XX[15]	46,XX[20]	0	89	-	0	41
12	RCMD	47,XY,+8[8]/46,XY[12]	46,XY[20]	0	80	13	0	50
13	RCMD	47,XX,+8[5]/46,XX[26]	46,XX[15]	0	82	3	0	100
14	RCMD	47,XY,+8[13]/46,XY[7]	46,XY[20]	0	78	17	0	70
15	RCMD	47,XX,+8[20]	46,XX[20]	2	92	60	-	-
16	RCMD	46,XX,del(5)(q14)[15]/47,XX,+8[2]	46,XX[15]	0	87	5	0	30
17	RCMD	47,XY,+8[8]/46,XY[15]	46,XY[20]	0	93	24	0	100
18	RAEB-1	47,XX,+8[9]/47,sl,i(17)(q10)[9]	No metaphases	0	90	73	0	76
19	RAEB-2	47,XY,+8[7]/46,XY[13]	46,XY[20]	0	93	43	0	53
20	RAEB-2	47,XX,+8[2]/46,XX[18]	46,XX[20]	0	89	6	0	72
21	RAEB-2	45,X,-Y[8]/46,X,-Y,+8[5]	46,X,-Y,+8[13]/46,XY[7]	0	96.7	47	0	54
22	MDS-U	47,XY,+8[19]/46,XY[1]	46,XY[20]	10	76	67	0	65
	**MDS/MPN**							
23	RARS-T	47,XX,+8[4]/46,XX[23]	46,XX[15]	0	93	-	0	31
24	RARS-T	47,XY,+8[3]/46,XY[17]	46,XY[15]	0	87	8	0	36
25	CMML	47,XY,+8[20]	46,XY[15]	8	84.7	-	0	80
26	CMML	**47,XY,+8[15]/46,XY[5]**	**47,XY,+8[2]/46,XY[48]**	**28**	**93**	**-**	**60**	**100**
	**AML**							
27	AML-MDRC	47,XY,+8[2]/46,XY[2]	46,XY[15]	0	92	7	0	41
28	AML NOS	No metaphases (FISH+8, 70%)	46,XX[15]	0	89	-	-	-
29	AML-MDRC	47,XY,+8[20]	46,XY[15]	0	95	58	0	83
30	APL	47,XX,+8,t(15;17)(q22;q12)[15] /46,XX [5]	46,XX[15]	0	93	-	0	100
31	AML NOS	48,XY,+8,+8[18]	46,XY[15]	0	93.8	-	0	100
32	AML-MDRC	46,XY,-5,del(7)(q11q35),+8,der(17)t(5;17)(p11;p11)[20]	No metaphases	0	84.8	-	0	100

**Abbreviations:** +8, trisomy 8; PB, peripheral blood; PHA, phytohemagglutinin; RA, refractory anemia; RCUD, refractory cytopenia with unilineage dysplasia; RCMD, refractory cytopenia with multilineage dysplasia; RAEB, RA with excess of blasts; MDS-U, myelodysplastic syndrome unclassified; RARS-T, RA with ringed sideroblasts and thrombocytosis; CMML, chronic myelomonocytic leukemia; AML, acute myeloid leukemia; AML-MDRC, AML with myelodysplasia-related changes; AML NOS, AML not otherwise specified; APL, acute promyelocytic leukemia. In bold patient with constitutional trisomy 8 mosaicism.

Among the ten patients with other myeloid neoplasms carrying +8, neither patients with RARS-T nor AML ones presented +8 on CD3+ lymphocytes and oral mucosa cells, while one of CMML patients showed trisomy 8 on both of them (CD3+ lymphocytes and oral mucosa cells).

For the healthy controls, the median of CD3+ cells with trisomy 8 was 1.3% and no cell from mucosa samples showed trisomy 8.

### Outcome analysis

The data of twenty-one patients with MDS and +8 were available for Kaplan-Meier analysis. Twelve patients died and five evolved to AML with a median follow up of 38.2 months (range, 2.6 to 92.3 months). The median OS and median time to AML transformation for MDS with isolated +8 were 85.9 and 2.8 months, respectively. No statistically significant differences in median OS were found between MDS with isolated +8 and MDS with +8 and another additional aberration.

## Discussion

MDS are associated with clonal cytogenetic abnormalities in around 50% of patients [2] being trisomy 8 the most common chromosome gain. According to the IPSS-R, isolated trisomy 8 is included in the intermediate cytogenetic risk group [4]. The current analysis with 22 patients diagnosed of MDS with isolated +8 and selected to be alive at the inclusion moment showed a longer overall survival (median, 85.9 months) than expected. However, in our previous study of 72 MDS with isolated +8 patients from GESMDregistry, the median overall survival was 34.3 months [3], demonstrating the intermediate risk confered by trisomy 8 to MDS and in agreement with IPSS-R. In contrast to other recurring chromosomal alterations, isolated +8 is not considered presumptive evidence of MDS when minimal morphological criteria are lacking [2]. This is in part because +8 may be derived from a constitutional 8 mosaicism. Furthermore, the incidence of cT8M among general population is very low [6,7]. In accordance, none of our healthy controls showed trisomy 8 by FISH. In 2002, Maserati *et al*. reported that +8 in myelodysplasia and acute leukemia is constitutional in 15–20% [9]. They had analyzed 13 cases of different myeloid neoplasms (including seven MDS) and 1 case of acute lymphoblastic leukemia and reported a cT8M in two of them after applying conventional cytogenetics from PB PHA-stimulated cultures. Nevertheless, in that study the cT8M was confirmed on a skin fibroblasts culture in only one MDS patient. Some other previous studies to determine lineage involvement in MDS, demonstrated that +8 was only found in myeloid lineage (granulocytes, monocytes and erythroblasts) [11–16]. These studies did not analyze non-hematopoietic cells because of their different aim. We evaluated the presence of +8 in 32 patients with different myeloid neoplasms (22 MDS, 2 RARS-T, 2 CMML and 6 AML). In all but one patient, we observed the +8 in myeloid cells and ruled it out in CD3+ lymphocytes and mucosa cells by FISH. Regarding the remaining patient, with +8 in both lymphocytes and mucosa cells, we could consider this alteration as constitutional. We believe that G-banding cytogenetics from PB PHA-stimulated cultures is not useful to discard cT8M, because myeloid cells present in these samples may also divide, giving a false positive result. In fact in our series, karyotype of PB showed +8 in 3 MDS patients but none of them presented +8 in oral mucosa samples. Hence, we consider it mandatory to apply FISH on isolated CD3+ lymphocytes as well as on non-hematological cells as oral mucosa ones for mosaicism studies. In the present project, the study of mucosa cells helps to rule out the germinal nature of trisomy 8 in those cases with residual positive CD3+ cells from samples with low purity. Non-use of the FISH technique on non-hematological cells probably explains the higher cT8M incidence reported from Maserati analyses in a short series with only 7 MDS patients [9]. Moreover, it is interesting to point out that the CMML patient with constitutional +8 had been diagnosed with a Behçet syndrome. Curiously the association between the presence of a cT8M and increased risk of developing Behçet syndrome [22] as well as a high risk of developing myeloid neoplasms [7,8,23], have already been referred.

Another argument used against the value of +8 to diagnose MDS is the possible presence of +8 as a seemingly clonal aberration in aplastic anemia (AA), which may disappear after immunosuppressive treatment [24]. Also Maciejewsky et al. have described a clonal evolution to MDS as a late complication of AA [25]. Thus, +8 in the absence of unequivocal dysplasia, would not be of help to differentiate hypocellular MDS from AA, entities that have been suggested to share similar pathogenic process for bone marrow hypocellularity [26]. Furthermore, a significant response rate of MDS with +8 to immunosuppressive therapy is well known [27].

In summary, our study confirms that cT8M should be ruled out using FISH on CD3+ lymphocytes and on non-hematological cells such as oral mucosa ones in MDS, and to the best of our knowledge, is the first study performed under these conditions. Besides this, our results suggest that trisomy 8 is acquired in almost all MDS, and probably, isolated +8 should be considered with enough evidence to diagnose MDS in normo and hypercellular bone marrow cases. Studies with longer series are needed for more decisive conclusions.
